# Epidemiology of the Microsporidium *Nosema ceranae* in Four Mediterranean Countries

**DOI:** 10.3390/insects13090844

**Published:** 2022-09-16

**Authors:** Clara Jabal-Uriel, Laura Barrios, Anne Bonjour-Dalmon, Shiran Caspi-Yona, Nor Chejanovsly, Tal Erez, Dora Henriques, Mariano Higes, Yves Le Conte, Ana R. Lopes, Aránzazu Meana, Maria Alice Pinto, Maritza Reyes-Carreño, Victoria Soroker, Raquel Martín-Hernández

**Affiliations:** 1 Instituto Regional de Investigación y Desarrollo Agroalimentario y Forestal de Castilla La Mancha (IRIAF), CIAPA de Marchamalo (Guadalajara, Spain), 19180 Marchamalo, Spain; 2 Unidad de Estadística, Centro Nacional de Investigaciones Científicas, 28006 Madrid, Spain; 3 INRAE, Unité de Recherche Abeilles et Environnement, National Institute for Agricultural, Food and Environmental Research, 84000 Avignon, France; 4 Mina and Aberhard Gudman Faculty of Life Sciences, Bar Ilan University, Ramat Gan 5290002, Israel; 5 Department of Entomology, Agricultural Research Organization, The Volcani Institute, Rishon LeZion 7505101, Israel; 6 Department of Environmental Economics and Management, The Robert H. Smith Faculty of Agriculture, Food and Environment, The Hebrew University of Jerusalem, Jerusalem 7610001, Israel; 7 Centro de Investigação de Montanha, Instituto Politécnico de Bragança, 5300-253 Bragança, Portugal; 8 Laboratório Associado Para a Sustentabilidade e Tecnologia em Regiões de Montanha (SusTEC), Instituto Politécnico de Bragança, Campus de Santa Apolónia, 5300-253 Bragança, Portugal; 9 Departamento de Sanidad Animal, Facultad de Veterinaria, Universidad Complutense, 28040 Madrid, Spain; 10 Instituto de Recursos Humanos para la Ciencia y la Tecnología, Fundación Parque Científico y Tecnológico de Castilla-La Mancha, 02006 Albacete, Spain

**Keywords:** climate, beekeeping management, honeybee, parasitism, *Apis mellifera*, colony losses

## Abstract

**Simple Summary:**

*Nosema ceranae* is a highly prevalent intracellular parasite of honey bees’ midgut worldwide. There is a lack of studies addressing the influence of climatic and beekeeping factors on the dynamics of its infection. A long-term study has been carried out in six apiaries in four Mediterranean countries (France, Israel, Portugal, and Spain), monitoring a total of 103 colonies. The lowest prevalence of infection was observed in mainland France, while the highest percentage of infected honey bees per colony was detected in Israel. The location and beekeeping management were shown to influence the infection levels. The percentage of infected honey bees negatively affected the colony strength in the apiaries located in Spain and mainland Portugal, whereas queen replacement had a positive effect on these same apiaries, reducing infection levels. The highest colony losses occurred in mainland France, which had the lowest levels of *N. ceranae*. It was followed by Spain, an apiary with high levels of *N. ceranae*, so no correlation between infection and mortality could be established. These results suggest that complementary studies on interactions with other pathogens and honey bee genetics are needed in order to develop management strategies for its control.

**Abstract:**

*Nosema ceranae* is a highly prevalent intracellular parasite of honey bees’ midgut worldwide. This Microsporidium was monitored during a long-term study to evaluate the infection at apiary and intra-colony levels in six apiaries in four Mediterranean countries (France, Israel, Portugal, and Spain). Parameters on colony strength, honey production, beekeeping management, and climate were also recorded. Except for São Miguel (Azores, Portugal), all apiaries were positive for *N. ceranae*, with the lowest prevalence in mainland France and the highest intra-colony infection in Israel. A negative correlation between intra-colony infection and colony strength was observed in Spain and mainland Portugal. In these two apiaries, the queen replacement also influenced the infection levels. The highest colony losses occurred in mainland France and Spain, although they did not correlate with the *Nosema* infection levels, as parasitism was low in France and high in Spain. These results suggest that both the effects and the level of *N. ceranae* infection depends on location and beekeeping conditions. Further studies on host-parasite coevolution, and perhaps the interactions with other pathogens and the role of honey bee genetics, could assist in understanding the difference between nosemosis disease and infection, to develop appropriate strategies for its control.

## 1. Introduction

The conservation of the abundance and diversity of insect pollinators is a decisive action to avoid the negative impact that the lack of these insects can have on agriculture, food production and security, and environmental sustainability. In this regard, managed honey bees are a suitable species that can easily be located in areas where they serve as a central pollination structure for a wide range of crops and a variety of wild flowers, which, in their absence, are not sustainable [1,2].

During the last few decades, there has been an alarming increase in honey bee colony losses where pathogens like *Varroa destructor* mites, the Microsporidia *Nosema* spp. and viruses contribute actively [3,4,5,6]. Pathogen spread within honey bee colonies is a dynamic process and is sometimes the result of the invasion of a new virulent pathogen and/or the combination with other pathogens and parasites that may be present in the colony [7,8,9].

Two Nosema species have been identified as honey bee pathogens: *Nosema apis* and *Nosema ceranae*, and nowadays, both species infect *Apis mellifera* colonies worldwide. However, *N. ceranae* is the species that has become one of the most prevalent honey bee pathogens globally [10,11,12]. This microsporidium is an obligate intracellular parasite of the ventricular cells of honey bees [13] and it is implicated in honey bee colony losses in some regions, especially in warm areas [12,14,15,16,17,18], probably due to the higher resistance of *N. ceranae* spores to heat and desiccation [19]. In *A. mellifera* honey bees, infection by this microsporidium induces damage to the ventriculus (midgut), which is the main site of nutrient absorption of the digestive tract and the target tissue of this pathogen. In this tissue, the infection causes degeneration of the epithelial cells, which are full of microsporidia in different stages of development (i.e., meronts, sporonts, sporoblasts, and spores), causing the weakening and death of infected honey bees [13,14]. In fact, the infection has been reported to shorten the lifespan [20,21,22], induce oxidative stress and changes in the metabolism and hormonal regulation of the honey bee host [21,23], or immune modulation [24,25,26,27], among other effects.

The prevalence of *N. ceranae* varies widely among locations. A common feature is that this pathogen is widely distributed regardless of the climatic conditions, ranging from desert climates [28,29,30] to very cold ones [31,32]. In some areas, this microsporidium is present in more than 50% of the colonies sampled [18,33,34,35,36,37,38,39], whereas in others the prevalence is lower than that percentage [40,41]. However, most studies consist of occasional surveys to determine the prevalence of infected colonies at a specific point in time [18,32,37,38,40,42,43,44,45,46]. Other studies carried out longitudinal surveys to determine the prevalence in selected apiaries and how it fluctuates across the study period. The findings varied among the studies, with countries such as Serbia [35], Germany [47], or New Zealand [48] showing a higher prevalence in spring and Uruguay showing a higher prevalence from the beginning of the winter until the end of the spring [49].

The prevalence of infection varies among colonies, and it is unclear how climate and beekeeping management affect the development of the pathogen and the resultant disease. Although variations at colony or apiary level throughout the year have been reported occasionally [14,50], comparisons among studies are difficult due to the diverse sampling and methodology employed, which hinder understanding of the true impact that climatic factors and beekeeping management have on the infection. Therefore, long-term research conducted using standardized protocols is needed to investigate these issues. To that end, this study aimed to compare *Nosema* spp. infection and its development in apiaries with very different climates and beekeeping practices during a 2-year period in four Mediterranean countries.

## 2. Materials and Methods

### 2.1. Study Design

**Figure 1:**
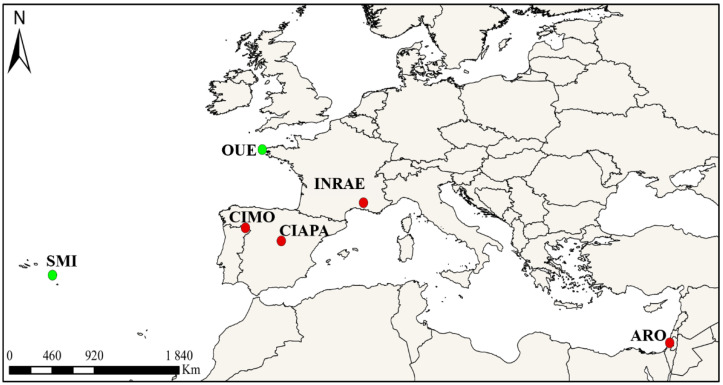
Apiary locations: Bragança, Portugal (CIMO), Fuente la Higuera, Spain (CIAPA), Avignon, France (INRAE), Zrifin, Israel (ARO), Ouessant Island, France (OUE), and São Miguel Island, Azores, Portugal (SMI). Red—*V. destructor* present, Green—*V. destructor* absent.

The study was carried out simultaneously in four Mediterranean countries: Portugal, Spain, France, and Israel. Six apiaries were selected to conduct the survey, namely, four locations where *V. destructor* is present, including CIAPA (Spain), INRAE (France), ARO (Israel), and CIMO (Portugal), and two others where *V. destructor* was absent, including the Ouessant (OUE, France) [51,52] and the São Miguel (SMI, Azores, Portugal) [53,54] islands. The exact locations of the apiaries and the total number of colonies involved in the study are shown in Figure 1 and Table 1. All colonies were prescreened to detect *N. ceranae* as described later. All the apiaries except São Miguel contained positive colonies at the beginning of the study. The São Miguel apiary was negative, although there is a history of *N. ceranae* presence on the island [55].

The colonies of the six apiaries were monitored for 20 months. Samplings started in February 2018 and ended in October 2019. On the islands (OUE and SMI), sampling started later in April 2018 due to weather conditions. Samplings were carried out every two months, except for December 2018 in most locations, due to the low temperatures. Colonies that died during the 20-month sampling period were replaced by spare colonies that were kept in the monitored apiaries under identical conditions (except in ARO) from the onset of the study.

### 2.2. Detection of N. ceranae Infection

In each sampling, adult workers were collected from each colony by brushing off the first comb with no brood surface from the brood chamber. This procedure was done to avoid collecting newborn workers. Samples were taken to the laboratory and kept at −80 °C for further analysis. All the apiaries were sampled using the same standardized protocol to allow comparisons.

The *Nosema* spp. infection was determined at apiary and intra-colony levels. At the apiary level, the presence of *N. ceranae* was determined for each colony from 60 adult workers that were processed as a pool. The pools prepared from each colony were placed in a sterile container (tubes or bags with a filter) and 15 mL or 6 mL of RNase-free water were added, respectively. The pools were homogenized using Stomacher^®^ (Qiagen, Hilden, Germany) and 180 µL of the macerates were transferred to 96-well plates (Qiagen, Hilden, Germany). The plates were shaken two times in a Tissuelyser (Qiagen, Hilden, Germany) for 1 min at 30 Hz, changing position between shacking rounds, followed by a short centrifugation. At ARO, the homogenization was performed with 50 mL tubes via Geno-grinder ^TM^ at 1550 rpm for 3 min, followed by a spin at 800 rpm 4 °C for 2 min. Afterwards, 50 µL of each homogenate was transferred to a 96-well plate, mixed with 50 µL TE buffer, and incubated at 95 °C for 20 min following the protocol [57] with a slight modification without the addition of Proteinase K [57].

At the intra-colony level, the prevalence of *N. ceranae* (percentage of bees infected per colony) was determined by individually analyzing 25 workers per colony, as described previously [14], which allowed us to establish the detection threshold at 4% (1 worker positive out of 25). Each worker was placed in a well of a 96-deep well plate with 500 µL of nuclease-free water and two steel beads. The plates were homogenized in a Tissuelyser (Qiagen, Hilden, Germany) for 1 min at 30 Hz four times or spun at 800 rpm 4 °C for 2 min in ARO. DNA extraction was performed in 50 µL of each homogenate, as explained above.

All PCRs were done as multiplex reactions with primers that allow for the detection of *N. ceranae*, *N. apis*, and an *A. mellifera* internal control (COI) in the laboratories of CIAPA (Spanish and Portuguese samples), INRAE (French samples), and ARO (Israeli samples) using a harmonized protocol [33]. PCR amplicons were revealed by agarose gels (French and Israeli samples) or by using the QIAxpert system (Spanish and Portuguese samples). Standard controls (*Nosema* spp. DNA and spores) were prepared by CIAPA and shared with INRAE and ARO to assure that the same specificity and sensitivity level were reached at the different facilities.

### 2.3. Beekeeping and Climatic Conditions

On every bimonthly visit to the apiary to collect samples, the colonies were inspected using the same standardized protocol and forms across countries to allow comparisons. Colony mortality, colony strength, colony management, the presence of pathogens (including Varroa levels and brood diseases), and control treatments were recorded. Assessment of colony strength was based on the type of hive, the number of combs covered by bees in the nest and supers, and the number of combs with brood. The percentage of area covered by honey bees was recorded on each side of the combs. Data were converted to the number of honey bees per colony, as indicated in the BEEBOOK [58]. In each brood comb, the area (in percentage) occupied by brood was visually estimated, and the quality of the brood was inspected for the presence of brood diseases. At every sampling date, the presence of the queen (color marked) was checked and, when the queen was not observed, the presence of recently laid eggs was verified. The presence of an unmarked queen in the colony was interpreted as a new queen born after a natural replacement, which was marked in situ according to the accepted international color code. Colony management consisted of recording any activity done. At the ARO apiary, a specific management of colonies was conducted in October–November 2018, in which all the queens were replaced by young mated queens and colonies were balanced through the exchange of brood combs and adults among the colonies of the apiary. Consequently, all colonies in 2019 were considered new ones.

In each apiary, the percentage of *V. destructor* infestation was determined during each sampling by using the sugar powder test, counting mite falls on 300 honeybees (limit of detection 0.3%) [59]. Additionally, *V. destructor* was controlled following national regulations (except in OUE and SMI, which were mite-free). In INRAE, two amitraz strips (Apivar^®^) as active ingredient (a.i.) per hive were used at the end of September each year. In ARO, 2–3 amitraz strips (Galvitraz^®^) per hive were applied in July and December each year. In CIMO and CIAPA, two amitraz strips (Apivar^®^ in 2018 and Apitraz^®^ in 2019) per hive were used in March and September 2018 and July 2019 (CIMO) or September 2019 (CIAPA); in March 2019, colonies were treated with thymol as a.i. using ApiLifeVar^®^ (CIMO) or Apiguard^®^ (CIAPA).

The presence of other pathogens in the colonies was inspected to detect any clinical signs of adult or brood diseases and was recorded in case of detection.

In addition, honey production was recorded annually by differences in the weight of honey combs from supers before and after honey extraction. The weather conditions were also recorded throughout the study by using meteorological stations in the mainland apiaries. Parameters recorded were mean, maximum, and minimum temperatures (°C); mean, maximum, and minimum relative humidity (%); mean wind speed (m/s); days with mean wind speed ≥6.4 m/s; and height of precipitation (mm).

### 2.4. Statistical Analysis

To determine whether there were any significant differences in the number of colonies infected by *N. ceranae* per apiary, all data from each site were analyzed together (cross-tabs, chi-square, with Monte Carlo correction, *p* < 0.0001). Differences in the intra-colony prevalence (percentage of honey bees infected per colony) among apiaries were analyzed by ANOVA. Homogeneity of variances was determined with a Levene test, and a post-hoc Games Howell or a Bonferroni test (depending on whether the variances were homogeneous or not) were used to compare among apiaries and/or sampling dates within the apiary. A Rho Spearman test was used to determine the correlation between the intra-colony infection level and the Varroa levels, the meteorological data, the colony strength data, and the honey production. The relationship between the levels of *N. ceranae* intra-colony infection in the colonies that replaced the queen or not was assessed using a Mann–Whitney U test. All *p*-values < 0.05 were considered significant, and all statistical analyses were carried out using the IBM SPSS Statistics V24 software by the Statistics Unit of the Scientific Computing Area at the SGAI-CSIC (Madrid, Spain).

## 3. Results

### 3.1. Nosema spp. Infection at the Apiary Level

Data on *Nosema* spp. infection was obtained from 103 colonies established at the beginning of the study (Table 1). The total number of colonies analyzed for each sampling round is shown in Table 2. As stated above, some colonies were added to replace the losses in order to monitor a sufficient number of colonies. Both *N. ceranae* and *N. apis* were analyzed in all the apiaries, but the latter was rarely found. Only one colony was positive for *N. apis* in April 2018 at the CIAPA, and therefore the following analyses were only performed on *N. ceranae*.

Most colonies were positive for *N. ceranae* in all apiaries throughout the study except on SMI, where no colony was positive. When we jointly analyzed all the data of infected colonies per apiary, the number of *N. ceranae*-positive colonies varied significantly among the apiaries (chi-square with Monte Carlo correction, *p* < 0.0001). All the colonies located in the CIAPA, ARO, and CIMO apiaries were positive for *N. ceranae* at the onset of the study and remained so nearly across the entire study period (February 2018–October 2019), and only on rare occasions, the microsporidium was not detected (Table 2). In contrast, *N. ceranae* was not detected in most of the INRAE colonies at the onset of the study, although the number of positive colonies increased in the following samplings, up to 66.67% in April 2018. From this moment on, the number of positive colonies was below 23%, except in August in both sampling years (50% and 60%, respectively). In the case of the OUE apiary, an intermediate pattern was observed, as all the colonies were positive at the beginning of the study and this was maintained during the first year with a low decrease (90% in October 2018), while in the second year, the number of *N. ceranae*-negative colonies decreased.

### 3.2. N. ceranae Infection at Intra-Colony Level

**Figure 2:**
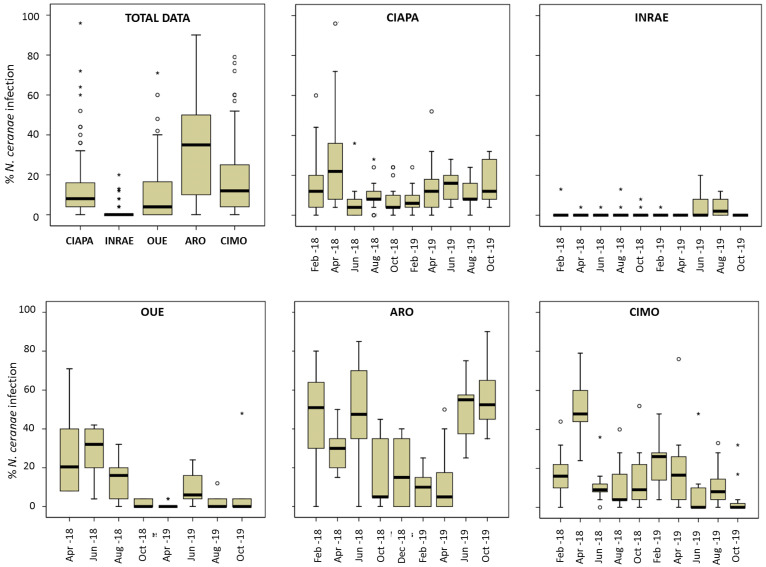
*N. ceranae* intra-colony level of infection. Total data per apiary and data per each apiary and sampling round. Data shows percentage of infected workers. Black line represents the median, while the box represents 50% of observations. Outliers are shown as dots and asterisks.

A total of 13,907 individual honey bees were screened for *N. ceranae* during the study (Table 3). The percentage of infected honey bees per colony (intra-colony prevalence) was also significantly (ANOVA; *p* < 0.0001) different among apiaries (Figure 2) and it varied across time (Figure 2), especially in ARO and CIMO. ARO had the highest mean prevalence (Table 3; Figure 2), with 32.39%, and it was significantly different from the other apiaries (Games–Howell test; *p* < 0.0001). ARO was followed by CIMO (17.17%), CIAPA (13.30%), and OUE (11.11%), and these three apiaries were not significantly different from each other (Games–Howell test; *p* > 0.05). Finally, INRAE had the significantly (Games–Howell test; *p* < 0.0001) lowest mean level of intra-colony infection (1.37%).

Regarding *N. apis*, only 14 out of the 13,907 honey bees analyzed were positive (two honey bees in CIAPA and 12 in CIMO), mostly found in coinfection with *N. ceranae* (12 honey bees coinfected). Thus, the percentage of infection by this species is not included.

In all apiaries, there were colonies with no infected honey bees at some point in time (Supplementary Table S1). On the other hand, the highest level of infection was detected in one colony from CIAPA (96%) in April 2018. In this apiary, April was the month with the highest mean intra-colony prevalence (27.64%; Figure 2, Supplementary Table S1) and the mean values kept similar and below 16% from that moment on until the end of the study (October 2019). The colonies of CIMO showed a similar pattern. The maximum level of infection was also found in April 2018 (50%) and it decreased afterwards, maintaining prevalence values below 20% until February 2019 (24.35%), decreasing again (circa 10%) until the end of the study. The ARO apiary exhibited the highest mean intra-colony prevalence, which was above 30% in all sampling rounds, except between October 2018 and April 2019. From that moment on, the prevalence increased gradually, reaching the highest levels of infection in October 2019 (56%). The colonies of the OUE apiary started with a relatively high percentage of infected honey bees (April and June 2018, >20%), decreasing thereafter to remain at levels below 10% until the end of the study. The intra-colony prevalence in INRAE showed the lowest levels of infection throughout the entire study period, with no significant differences among the samplings, as the mean values were below 2% in most of the samplings, and only in June and August 2019 did it increase to 4%. The highest value (20%) was found in one colony in June 2019 and it became under the detection level in the following sampling round. The SMI was in stark contrast to the remaining apiaries, as no honey bees were detected as positive. The full set of results on the significant differences in the percentage of infected bees for each sampling round and apiary can be found in the Supplementary Table S2.

### 3.3. Varroa Destructor Levels

A Varroa test was performed only in the apiaries of CIMO, CIAPA, INRAE, and ARO as the islands of SMI and OUE were mite free. The infestation levels differed among apiaries, with the highest mean percentage found in CIMO (1.09%) and ARO (0.9%), and the lowest in CIAPA (0.18%; Table 4). It should be noted that, except for CIAPA, the maximum levels of Varroa were over 9% at INRA, ARO, and CIMO but in just one colony at each apiary. However, only 36 samples, out of the 419 analyzed, exceeded 2% (11 from INRA, 12 from ARO, and 13 from CIMO).

There was a positive correlation between the percentage of Varroa in the colony and the *N. ceranae* intra-colony infection level (Spearman’s Rho, 2-tailed; *p* < 0.005), when all the data were analyzed together. This correlation was maintained in the INRAE and ARO apiaries when analyzed separately (Spearman’s Rho, 2-tailed; *p* < 0.005) whereas in the CIMO and CIAPA apiaries, there was no correlation.

### 3.4. Climatic Conditions and N. ceranae Infection

**Figure 3:**
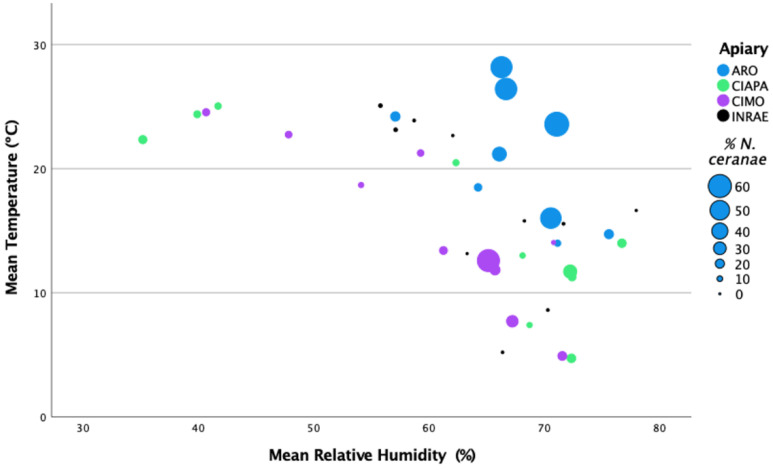
Representation of *N. ceranae* infection levels (percentage of infected honey bees) per apiary and sampling round in relation to the mean temperature (°C) and the mean relative humidity (%).

The ARO apiary was located in the warmest region with the highest mean temperature (22.16 °C) and mean relative humidity (67.17%). The INRAE apiary exhibited the highest mean precipitation (1.82 mm), whereas CIMO showed the highest mean wind speed (6.25 m/s) of all the studied apiaries (Figure 3; Supplementary Table S3).

The analysis between the mean percentage of intra-colony infection per apiary across samplings and the climatic conditions (monthly means) did not show significant correlations with the recorded parameters (Spearman’s Rho, 2-tailed; *p* > 0.05). Only the number of days with a wind speed higher than 6.4 m/s was positively correlated with *N. ceranae* levels (Spearman’s Rho, 2-tailed; *p* < 0.005), which could be related with to number of days that honey bees are inside the colony. However, these data were only recorded for the CIMO and INRAE apiaries, and this correlation could not be confirmed at the other locations.

When the mean temperature and mean relative humidity were represented together in relation to the *N. ceranae* infection levels, the highest levels were found at the highest mean temperature and mean relative humidity of over 65% (Figure 3). These values match the ARO apiary, which exhibited *N. ceranae* infection levels significantly higher than in the other apiaries (Games–Howell test; *p* < 0.0001) (see Section 3.2: *N. ceranae* infection at intra-colony level).

### 3.5. Colony Strenght and N. ceranae Infection Levels

**Figure 4:**
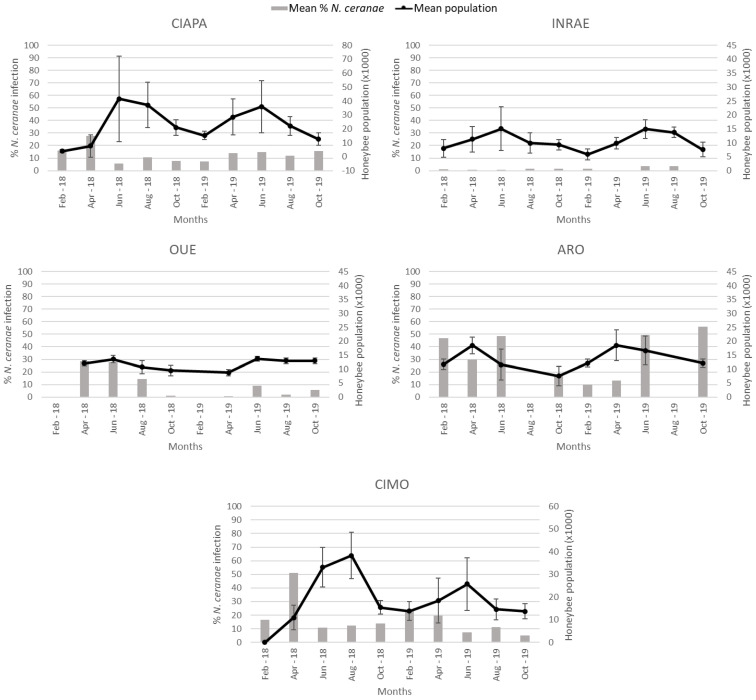
Mean percentage of *N. ceranae* infection (grey columns) and mean colony strength and standard deviation (black line) per apiary during the study period. Colony strength is represented in number of honeybees (thousands). In OUE and ARO, there was no data collection in February and August, respectively, due to weather conditions.

There was considerable variation in colony strength (number of adult honey bees per colony) both among and within the apiaries (Figure 4). CIAPA exhibited the highest mean (23,124.16) of colony strength and INRAE, the lowest (10,453.83; Table 5). ARO was the first apiary where the population began to increase in spring, reaching maximum values in April in both years. This pattern contrasted with that of the other apiaries, where colony strength peaked in the summer (Figure 4).

Colony strength, estimated at each sampling round, was analyzed to determine if there was a relationship with the percentage of honey bees infected by *N. ceranae* (Figure 4). When the data generated for each apiary were analyzed altogether, there was a positive correlation (r = 0.179) between the two variables (Spearman’s Rho; *p* < 0.0001). However, when the analysis was done individually for each apiary, a different pattern was observed. While in the CIAPA and CIMO apiaries, a significant negative correlation (r = −0.299 and r = −0.257, respectively) between the percentage of *N. ceranae* infection and colony strength was observed (Spearman’s Rho; *p* < 0.01), and in INRAE and OUE, the correlation was positive (Spearman Rho; r = 0.252 and r = 0.299, respectively; *p* < 0.01). In ARO, the correlation was not significant, although the trend was negative as in CIAPA and CIMO (Spearman’s Rho; *p* > 0.05). SMI was not included in the analysis because *N. ceranae* was not detected in any colony across time.

### 3.6. Honey Production and N. ceranae Infection Levels

The honey production (Kg) per colony was recorded only in the continental apiaries (Table 6). CIMO and CIAPA had the highest mean of honey production (29.30 Kg and 27.64 Kg, respectively), followed by ARO (18.15 Kg) and INRAE (9.16 Kg, data available only for 2019), which is consistent with colony strength. There was no significant correlation between *N. ceranae* intra-colony infection and honey production (Spearman’s Rho; *p* > 0.05).

### 3.7. Queen Replacement and N. ceranae Infection Levels

In each sampling round, the presence of the queen was checked. Thus, every queen replacement was recorded except on the islands, in which those data were not available.

**Figure 5:**
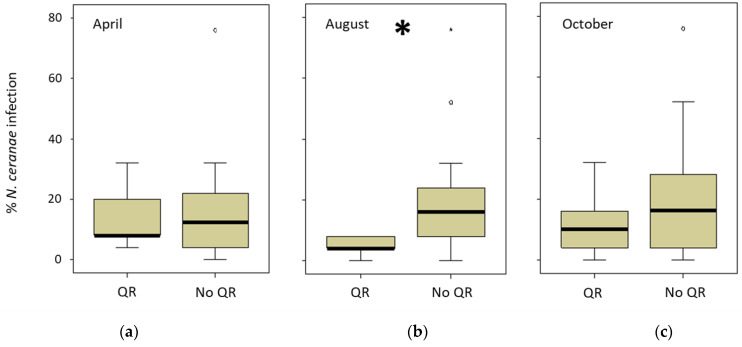
Percentage of intra-colony infection in CIAPA and CIMO colonies in April 2019. The comparison was made between colonies when the queen replacement (QR) was detected in (**a**) April 2019 (*n* = 5), (**b**) August (*n* = 5), or (**c**) October (*n* = 14) versus the colonies that did not replace the queen (No QR) for the same periods (*n* = 16; *n* = 23; *n* = 14, respectively). * Denotes significant differences (*p* < 0.05).

The ARO apiary had a special colony management in autumn in which comb number was equalized and the queens were artificially replaced. INRAE only registered one natural queen replacement (June 2018), whereas CIMO registered seven (three colonies out of 15 in 2018 and four out of 12 in 2019) during the study period. However, in the CIAPA apiary, all the colonies (*n* = 22) replaced their queen (naturally) at least once between March and September 2018, and two colonies even did it three times. The queen replacement was lower in the following year (five colonies out of 16). Therefore, the relationship between the queen replacement and the percentage of infected honeybees per colony was analyzed only for the CIAPA and CIMO apiaries (Supplementary Table S4). To do this, the percentage of honeybees infected in the sampling previous to the queen replacement (detected in April, August, or October 2018) was compared to the percentage of honey bees infected in the following spring (April 2019). The comparisons were made between two groups: one including the colonies that changed the queen and another group of colonies that did not change the queen. The only significant (Mann–Whitney test; *p* < 0.05) difference was found in the levels of intra-colony infection that were lower in the colonies that had replaced the queen in the previous summer (detected in August 2018) as opposed to those that had not changed it (Figure 5).

### 3.8. Colony Mortality and N. ceranae Infection Levels

The mortality of colonies was recorded across the 20-month period (Table 7). The highest percentage of losses was observed in INRAE (47.6%) and CIAPA (40.9%), and the lowest on the islands, with one colony lost in OUE and none in SMI. The level of *N. ceranae* infection of a colony could not be correlated with its mortality. Only one colony (in CIAPA) had 96% of honey bees infected (Supplementary Table S1), which died a month later after infection assessment. When *N. ceranae* intra-colony infection in the two months prior to colony death was analyzed, a large proportion of the dead colonies were found to have a level of infection greater than or equal to 20% (Supplementary Table S4). Studying all the data together, 46% of the deceased colonies exceeded this value. However, this condition was not fulfilled in OUE or INRAE, so the data were analyzed by grouping the dead colonies from CIAPA, ARO, and CIMO. Thus, 76.5% of the colonies had ≥20% of honey bees infected in the previous two months (CIAPA: 77%, ARO and CIMO: 75%, respectively). Still, some colonies in CIMO or ARO reached over 75% of infection and did not die, having a remarkable decrease of infected honey bees at the following sampling round (below 40%; Supplementary Table S4).

## 4. Discussion

The prevalence of *N. ceranae* infection was determined at the apiary and intra-colony levels in six apiaries, covering a wide range of Mediterranean environmental and beekeeping conditions in a long-term study conducted on a large batch of data (103 colonies across 20 months; 13,907 individual worker honey bees). This allowed us to determine the differences in *N. ceranae* infection among the different environments, confirming that the epidemiology of the infection by the microsporidium varies geographically and temporally. *Nosema ceranae* infection was confirmed in all the apiaries studied except in SMI, indicating that the microsporidium is widely spread.

The number of colonies infected by *N. ceranae* in the INRAE (France) apiary was significantly lower than that of the other apiaries. In OUE (France), ARO (Israel), CIMO (Portugal), and CIAPA (Spain), a high number of positive colonies were detected in the four apiaries throughout the sampling rounds, although the intra-colony infection levels varied greatly among apiaries, with ARO exhibiting the highest percentage of infected honey bees. This finding could be partly explained by the climatic conditions at each site, which were different. However, in our study, there was no correlation between the climatic parameters alone and the infection levels, and just the number of days with high wind speed increased the percentage of bees infected per colony. Only a visual and non-significant correlation of the combined effect of high mean temperature and high mean relative humidity with higher levels of infection could be intuited. As opposed to our results, temperature and humidity were correlated positively with *N. ceranae* incidence (spore density) but negatively to *N. apis* incidence in Turkey [60] and the levels of *N. ceranae* were negatively correlated with high temperatures in Serbia, [61]. Another study in China also showed higher *N. ceranae* prevalence in the more humid regions (South) when compared with apiaries in dryer areas (North) [45]. Moreover, in a previous study carried out in CIAPA [14], monthly rainfall was positively correlated with the percentage of interior honey bees infected by *N. ceranae* and the percentage of foragers infected was negatively correlated with the mean maximum temperature. Therefore, our results suggest that other factors than climatic conditions could play a role in the prevalence of the *N. ceranae* infection.

A higher level of *N. ceranae* infection found in CIAPA, CIMO, and ARO (not statistically significant in the latter) was correlated to a lower adult honey bee population, as opposed to the positive correlation found in INRAE and OUE apiaries. The different relationships between those two groups in colony strength and the percentage of honey bees infected by *N. ceranae* could be explained by the low level of infection in INRAE (for all samplings) and OUE (2019). On the other hand, the relationship observed in Iberia (CIAPA and CIMO) and Israel (ARO) between the *N. ceranae* infection level and colony strength seem to confirm previous findings in Spain [14,62,63], where infection has been shown to be detrimental to honey bee colonies.

Beekeeping management in ARO was different from that of the other apiaries. Given that this apiary had the highest levels of infection but had only 20.1% colony mortality, it could indicate that the management was able to control the mortality associated with the high percentage of infected honey bees per colony [64]. Beekeeping management, and, in particular, queen replacement with a younger queen, has been identified as a biotechnical method to control nosemosis [65,66]. This recommendation is consistent with our results as the levels of intra-colony infection in spring were lower in colonies that had replaced the queen in the previous summer. Other authors found that one-year-old queens are able to compensate for the effects of *Nosema* infection, with this ability gradually decreasing in subsequent years [66]. As well, the infection by *N. ceranae* has been reported to reduce honey production [67], although in this work we could not establish any correlation between the intra-colony infection level and the honey produced.

The highest colony mortality was recorded in INRAE, which had the apiary with the lowest *N. ceranae* infection levels. It is very likely that the microsporidium infection was not related to the mortality in this apiary and that other biotic and abiotic stressors contributed to colony mortality and possibly to the lower honey production. One of these biotic stressors could be Varroa. However, this trial was not designed to monitor Varroa levels but only to detect high infestations so that control measures could be taken. Moreover, infestation levels were below 2% in most of the cases, so their influence on the colony mortality seems to be limited. Despite this, a significant correlation between the percentage of honey bees infected by *N. ceranae* and *V. destructor* levels was found in two apiaries (INRAE and ARO) out of four analyzed. The correlation between the two pathogens is unclear as, such as in this study, there are studies that both confirm [68] and fail to find a correlation [15,69]. In addition, the Varroa treatments made in each country could have any influence, as oxalic acid has shown an effect on *N. ceranae* infection [70] and, conversely, infection by Microsporidium could reduce the efficacy of the results [71]. Hence, it is possible that there are factors yet to be determined that may influence the interaction between them. It is well known that *V. destructor* is an effective vector for *Deformed wing virus* (DWV), which was frequently detected in INRAE (unpublished data) and probably impacted colony losses in this apiary. On the other hand, the apiaries located on the *V. destructor*-free islands registered the lowest mortality levels. Thus, the presence of the mite seems to complicate the pathological consequences caused by other pathogens [68,72], as the intensity of these pathogens (*N. ceranae* and viruses) seems to increase when they appear together [73]. The overall mortality at the CIAPA (40.9%), CIMO (26.7%), and ARO (20.1%) apiaries could be considered high, when compared to the percentage of winter mortality rate of 10.7% reported in 35 countries for the same time frame (2018–2019) [74], although overall mortality is expected to be higher than during overwintering. In this way, although no infection rate could be established as a marker for colony mortality, it is possible that the *N. ceranae* infection plays a role in the losses, as this microsporidium can cause the death of the infected honey bees [14], impacting on the viability of the colonies [14,15,18,29,75,76,77]. High levels of infection have been reported as a cause of colony losses [14,62]. In our study, the 76.5% of dead colonies from CIAPA, ARO, and CIMO had ≥20% of honey bees infected in the previous two months. These high levels recorded in our study in CIAPA, ARO, and CIMO imply a sustained stress on the colonies. It has been published that an infectious disease causing mortality in foraging bees is very dangerous for the survival of a honey bee colony [78]. In addition, *Nosema ceranae* infection decreases the energy status of honey bees, leading to changes in their foraging behavior, which have a strong adverse effect on energetic gain efficiency [79]. This will have an effect on food availability and it could lead to colony failure when the forager mortality rate reaches a critical threshold [80].

In addition to the presence of *N. ceranae* infection, other stressors such as pathogens and pollutants have been shown to impact colony losses [81,82,83,84]. In fact, coinfection of microsporidia with very common viral pathogens might contribute to the death of the colony, even for asymptomatic infections [9,85]. Moreover, stress factors affecting honey bee immunity may trigger latent viral infections to become overt infections. Thus, insecticides [83,86,87] or infestation with other pathogens or parasites like *V. destructor* may activate dormant virus infections in the colony and even promote selection of virulent strains, such as in the case of DWV [88,89,90,91]. Moreover, the host genetics may influence the development (or the consequences) of the infection since the levels of *N. ceranae* infection have been noticed to be significantly different between lineages and colonies for both Russian and Italian honey bees, and in the case of the Russian lineage, the patriline-based variance was also found to be significant [92]. Therefore, the differences in the dynamics and the levels of *N. ceranae* infection here observed could also be related to the different honey bee lineages studied. Nevertheless, it has been suggested that genetic diversity cannot compensate and reduce alone the levels of infection [61].

Our results obtained from apiaries set in several Mediterranean countries confirm the high variability of *N. ceranae* prevalence. *Nosema ceranae* has been shown to be a very adaptable parasite and the effects of beekeeping management (such as queen replacement) were found to influence the prevalence, probably more linked with the colony biology adaptations than with the parasite itself. Clinical signs related to *N. ceranae*’s high prevalence and climatic conditions, such as lower honey production rate, depopulation, and death, were detected in some Mediterranean countries during initial reports [14,15,29,62,93] when the epidemic wave was at its highest. The comparison among different countries after several years in similar conditions, together with the fact that some highly infected colonies did not die, seems to indicate the initial lethal effect of the new parasite for *Apis mellifera* is downgrading, as coevolution contributes to establishing host–parasite equilibrium. Further studies on this topic, including the role of honey bee genetics, could assist in understanding the difference between nosemosis disease and infection, in a rather similar way to varroosis and Varroa detection.

## Figures and Tables

**Table 1:** Location of the apiaries, number of colonies (*n* = 103) and mitochondrial DNA (MtDNA) lineages of honey bees.

Country	Apiary Name	Coordinates (Latitude/Longitude)	Locality	Total No. Colonies per Apiary	MtDNA Lineages ^1^ [56]
Spain	CIAPA	40.751389/−3.303889	Fuente la Higuera de Albatages	22	A and M
France	INRAE	43.946941/4.862223	Avignon	21	C
France	OUE	48.477008/−5.067211	Cadoran	11	M
Israel	ARO	31.966979/34.843588	Zrifin	24	C
Portugal	CIMO	41.808791/−6.711865	Bragança	15	A and M
Portugal	SMI	37.752648/−25.588381	Ribeira do Guilherme	10	A

^1^ A–African lineage; M–western European lineage; C–eastern European lineage.

**Table 2:** Percentage of colonies positive for *N. ceranae* per sampling and apiary and number of positive colonies out of sampled colonies in parenthesis. SMI had 10 colonies where *Nosema* spp. were never detected. ND: Not detected.

	2018					2019				
	February	April	June	August	October	February	April	June	August	October
CIAPA	100.00 (15/15)	100.00 (22/22)	95.24 (20/21)	100.00 (17/17)	100.00 (16/16)	100.00 (16/16)	100.00 (16/16)	100.00 (14/14)	100.00 (13/13)	100.00 (13/13)
INRAE ^1^	7.69 (1/13)	66.67 (4/6)	14.29 (1/7)	50.00 (5/10)	22.22 (2/9)	6.67 (1/15)	ND (0/14)	20.00 (3/15)	60.00 (9/15)	20.00 (3/15)
OUE	-	100.00 (10/10)	100.00 (10/10)	100.00 (10/10)	90.00 (9/10)	-	30.00 (3/10)	100.00 (10/10)	80.00 (8/10)	40.00 (4/10)
ARO	100.00 (14/14)	100.00 (14/14)	100.00 (13/13)	-	100.00 (9/9)	100.00 (12/12)	91.00 (10/11)	100.00 (11/11)	-	100.00 (10/10)
CIMO	100.00 (15/15)	100.00 (15/15)	100.00 (15/15)	86.67 (13/15)	86.67 (13/15)	100.00 (12/12)	100.00 (12/12)	63.64 (7/11)	100.00 (11/11)	54.55 (6/11)

^1^ Only data from colonies from which it was possible to collect samples are shown. Data not available.

**Table 3:** Intra-colony prevalence at each apiary. Percentage of *N. ceranae* infected honey bees per colony during the study. ND: Not detected (<4%).

	CIAPA	INRAE	OUE	ARO	CIMO	SMI
No. bees analysed	4075	2369	1993	1888	3102	480
Mean (%)	13.30	1.37	11.11	32.39	17.17	ND
Std. Dev.	14.12	3.58	15.49	24.29	17.93	ND
Median	8.00	0.00	4.00	35.00	12.00	ND
Max.	96.00	20.00	70.83	90.00	79.17	ND
Min.	ND	ND	ND	ND	ND	ND

**Table 4:** Statistics for the percentage of *Varroa destructor* in 300 honey bees per apiary across sampling rounds (*n* = 419). Islands are not included as they were the mite free.

Apiary	CIAPA	INRA	ARO	CIMO
N	132	102	81	104
Mean	0.18%	0.71%	0.9%	1.09%
Std. Dev.	0.54	1.58	1.71	1.73
Min.	0%	0%	0%	0%
Max.	3.7%	9.7%	9.61%	9.61%

**Table 5:** Estimation of colony strength for each apiary. Values represent an estimation of the number of honey bee adults.

	CIAPA	INRAE	Ouessant	ARO	CIMO	SMI
No. samples analyzed ^1^	163	119	80	107	132	80
Mean	23,124.16	10,453.83	11,778.84	13,352.70	18,717.05	15,870.75
Median	17,850	9808.40	12,610.80	13,078.13	16,590.00	16,800.00
Std. Dev.	18,946.79	4609.50	2238.07	4984.24	13,069.74	8216.15
Minimum	1050	2802	7006	5231	2100	6300
Maximum	91,560	32,386	14,012	22,494	60,060	33,600

^1^ Samples correspond to colonies analyzed across sampling rounds.

**Table 6:** Average honey production per colony (in Kg) at each apiary during the study period. Islands are not included. Data not available.

Apiary	No. of Colonies	2018	2019	Total Mean	Median	Std. Dev.	Min.	Max.
CIAPA	25	26.7	33.86	29.30	30.03	13.93	6.64	52.94
INRAE	15	-	9.16	9.16	9.00	5.78	0.00	17.60
ARO	52	17.50	18.93	18.15	18.18	10.62	0.00	35.90
CIMO	29	33.40	21.46	27.64	26.00	18.85	0.00	63.10

**Table 7:** Total number of colonies studied per apiary and colony mortality.

Apiary	No. Colonies in the Study	No. of Dead Colonies	Percentage of Losses	No. of Dead Colonies ≥20% of Intra-Colony Infection ^2^
CIAPA	22	9	40.9%	7
INRAE	21	10	47.6%	0
OUE	11	1	9.1%	0
ARO	24	5 ^1^	20.1%	3
CIMO	15	4	26.7%	3
SMI	10	0	0.0%	0

^1^ One colony with no data of *N. ceranae* intra-colony infection in the two months prior to death. ^2^ Data corresponds to two sampling rounds prior to death.

## Data Availability

The data presented in this study are available on request from the corresponding author.

## Supplementary Materials

The following supporting information can be downloaded at: https://www.mdpi.com/article/10.3390/insects13090844/s1, Table S1: Mean levels of infection per month in each apiary; Table S2: Statistical differences of infected honey bees for each sampling and apiary; Table S3: Climatic conditions recorded per in mainland apiaries; Table S4: Colony losses and queen replacement per apiary.

## References

[1] FAO, IZSLT, Apimondia, CAAS (2021). Good Beekeeping Practices for Sustainable Apiculture.

[2] Bishop J., Garratt M.P.D., Nakagawa S. (2022). Animal Pollination Increases Stability of Crop Yield across Spatial Scales. Ecol. Lett..

[3] vanEngelsdorp D., Meixner M.D. (2010). A Historical Review of Managed Honey Bee Populations in Europe and the United States and the Factors That May Affect Them. J. Invertebr. Pathol..

[4] Meixner M.D., Costa C., Kryger P., Hatjina F., Bouga M., Ivanova E., Büchler R. (2010). Conserving Diversity and Vitality for Honey Bee Breeding. J. Apic. Res..

[5] Chauzat M.P., Carpentier P., Madec F., Bougeard S., Cougoule N., Drajnudel P., Clément M.C., Aubert M., Faucon J.P. (2010). The Role of Infectious Agents and Parasites in the Health of Honey Bee Colonies in France. J. Apic. Res..

[6] Neov B., Georgieva A., Shumkova R., Radoslavov G., Hristov P. (2019). Biotic and Abiotic Factors Associated with Colonies Mortalities of Managed Honey Bee (*Apis Mellifera*). Diversity.

[7] Mcmahon D.P., Fürst M.A., Caspar J., Theodorou P., Brown M.J.F., Paxton R.J. (2015). A Sting in the Spit: Widespread Cross-Infection of Multiple RNA Viruses across Wild and Managed Bees. J. Anim. Ecol..

[8] Ryabov E.V., Childers A.K., Lopez D., Grubbs K., Posada-Florez F., Weaver D., Girten W., vanEngelsdorp D., Chen Y., Evans J.D. (2019). Dynamic Evolution in the Key Honey Bee Pathogen Deformed Wing Virus: Novel Insights into Virulence and Competition Using Reverse Genetics. PLoS Biol..

[9] Borba R.S., Hoover S.E., Currie R.W., Giovenazzo P., Guarna M.M., Foster L.J., Zayed A., Pernal S.F. (2022). Phenomic Analysis of the Honey Bee Pathogen-Web and Its Dynamics on Colony Productivity, Health and Social Immunity Behaviors. PLoS ONE.

[10] Martín-Hernández R., Bartolomé C., Chejanovsky N., Le Conte Y., Dalmon A., Dussaubat C., García-Palencia P., Meana A., Pinto M.A., Soroker V. (2018). *Nosema Ceranae* in *Apis Mellifera*: A 12 Years Postdetection Perspective. Environ. Microbiol..

[11] Grupe A.C., Alisha Quandt C. (2020). A Growing Pandemic: A Review of *Nosema* Parasites in Globally Distributed Domesticated and Native Bees. PLoS Pathog..

[12] Marín-García P.J., Peyre Y., Ahuir-Baraja A.E., Garijo M.M., Llobat L. (2022). The Role of *Nosema Ceranae* (Microsporidia: Nosematidae) in Honey Bee Colony Losses and Current Insights on Treatment. Vet. Sci..

[13] Higes M., García-Palencia P., Martín-Hernández R., Meana A. (2007). Experimental Infection of *Apis Mellifera* Honeybees with *Nosema Ceranae* (Microsporidia). J. Invertebr. Pathol..

[14] Higes M., Martín-Hernández R., Botías C., Bailón E.G., González-Porto A.V., Barrios L., Del Nozal M.J., Bernal J.L., Jiménez J.J., Palencia P.G. (2008). How Natural Infection by *Nosema Ceranae* Causes Honeybee Colony Collapse. Environ. Microbiol..

[15] Soroker V., Hetzroni A., Yakobson B., David D., David A., Voet H., Slabezki Y., Efrat H., Levski S., Kamer Y. (2011). Evaluation of Colony Losses in Israel in Relation to the Incidence of Pathogens and Pests. Apidologie.

[16] Bacandritsos N., Granato A., Budge G., Papanastasiou I., Roinioti E., Caldon M., Falcaro C., Gallina A., Mutinelli F. (2010). Sudden Deaths and Colony Population Decline in Greek Honey Bee Colonies. J. Invertebr. Pathol..

[17] Natsopoulou M.E., McMahon D.P., Doublet V., Bryden J., Paxton R.J. (2015). Interspecific Competition in Honeybee Intracellular Gut Parasites Is Asymmetric and Favours the Spread of an Emerging Infectious Disease. Proc. R. Soc. B Biol. Sci..

[18] Moeini S., Malekifard F., Tavassoli M. (2022). Identification of the *Nosema* Spp., a Microsporidian Parasite Isolated from the Honey Bees (*Apis Mellifera*) and Its Association with Honey Bee Colony Losses in Apiaries of Iran. J. Hell. Vet. Med. Soc..

[19] Fenoy S., Rueda C., Higes M., Martín-Hernández R., Del Aguila C. (2009). High-Level Resistance of *Nosema Ceranae*, a Parasite of the Honeybee, to Temperature and Desiccation. Appl. Environ. Microbiol..

[20] Alaux C., Brunet J.L., Dussaubat C., Mondet F., Tchamitchan S., Cousin M., Brillard J., Baldy A., Belzunces L.P., Le Conte Y. (2010). Interactions between *Nosema* Microspores and a Neonicotinoid Weaken Honeybees (*Apis Mellifera*). Environ. Microbiol..

[21] Dussaubat C., Brunet J.L., Higes M., Colbourne J.K., Lopez J., Choi J.H., Martín-Hernández R., Botías C., Cousin M., McDonnell C. (2012). Gut Pathology and Responses to the Microsporidium *Nosema Ceranae* in the Honey Bee *Apis Mellifera*. PLoS ONE.

[22] Martín-Hernández R., Botías C., Barrios L., Martínez-Salvador A., Meana A., Mayack C., Higes M. (2011). Comparison of the Energetic Stress Associated with Experimental *Nosema Ceranae* and *Nosema Apis* Infection of Honeybees (*Apis Mellifera*). Parasitol. Res..

[23] Vidau C., Diogon M., Aufauvre J., Fontbonne R., Viguès B., Brunet J.L., Texier C., Biron D.G., Blot N., Alaoui H. (2011). Exposure to Sublethal Doses of Fipronil and Thiacloprid Highly Increases Mortality of Honeybees Previously Infected by *Nosema Ceranae*. PLoS ONE.

[24] Antúnez K., Martín-Hernández R., Prieto L., Meana A., Zunino P., Higes M. (2009). Immune Suppression in the Honey Bee (*Apis Mellifera*) Following Infection by *Nosema Ceranae* (Microsporidia). Environ. Microbiol..

[25] Chaimanee V., Chantawannakul P., Chen Y., Evans J.D., Pettis J.S. (2012). Differential Expression of Immune Genes of Adult Honey Bee (*Apis Mellifera*) after Inoculated by *Nosema Ceranae*. J. Insect Physiol..

[26] Schwarz R.S., Evans J.D. (2013). Single and Mixed-Species Trypanosome and Microsporidia Infections Elicit Distinct, Ephemeral Cellular and Humoral Immune Responses in Honey Bees. Dev. Comp. Immunol..

[27] Lourenço A.P., Guidugli-Lazzarini K.R., de Freitas N.H.A., Message D., Bitondi M.M.G., Simões Z.L.P., Teixeira É.W. (2021). Immunity and Physiological Changes in Adult Honey Bees (*Apis Mellifera*) Infected with *Nosema Ceranae*: The Natural Colony Environment. J. Insect Physiol..

[28] Haddad N.J. (2014). First Detection of *Nosema Ceranae* in Jordan. Eur. Sci. J..

[29] Adjlane N., Haddad N. (2016). Effect of Some Honeybee Diseases on Seasonal Mortality of *Apis Mellifera* Intermissa in Algeria Apiaries. Proc. Zool. Soc..

[30] Ansari M.J., Al-Ghamdi A., Nuru A., Khan K.A., Alattal Y. (2017). Geographical Distribution and Molecular Detection of *Nosema Ceranae* from Indigenous Honey Bees of Saudi Arabia. Saudi J. Biol. Sci..

[31] Tokarev Y.S., Zinatullina Z.Y., Ignatieva A.N., Zhigileva O.N., Malysh J.M., Sokolova Y.Y. (2018). Detection of Two Microsporidia Pathogens of the European Honey Bee *Apis Mellifera* (Insecta: Apidae) in Western Siberia. Acta Parasitol..

[32] Ostroverkhova N.V. (2020). Prevalence Of *Nosema Ceranae* (Microsporidia) In The *Apis Mellifera Mellifera* Bee Colonies From Long Time Isolated Apiaries Of Siberia. Far East. Entomol..

[33] Martín-Hernández R., Botías C., Bailón E.G., Martínez-Salvador A., Prieto L., Meana A., Higes M. (2012). Microsporidia Infecting *Apis Mellifera*: Coexistence or Competition. Is *Nosema Ceranae* Replacing *Nosema Apis*?. Environ. Microbiol..

[34] Cepero A., Martín-Hernández R., Bartolomé C., Gómez-Moracho T., Barrios L., Bernal J., Teresa Martín M., Meana A., Higes M. (2015). Passive Laboratory Surveillance in Spain: Pathogens as Risk Factors for Honey Bee Colony Collapse. J. Apic. Res..

[35] Stevanovic J., Simeunovic P., Gajic B., Lakic N., Radovic D., Fries I., Stanimirovic Z. (2013). Characteristics of *Nosema Ceranae* Infection in Serbian Honey Bee Colonies. Apidologie.

[36] Stevanovic J., Schwarz R.S., Vejnovic B., Evans J.D., Irwin R.E., Glavinic U., Stanimirovic Z. (2016). Species-Specific Diagnostics of *Apis Mellifera* Trypanosomatids: A Nine-Year Survey (2007–2015) for Trypanosomatids and Microsporidians in Serbian Honey Bees. J. Invertebr. Pathol..

[37] Matthijs S., Waele D., Vandenberge V., Evers J., Brunain M., Saegerman C., De Winter P.J.J., Roels S., Graaf D.C., De Regge N. (2020). Nationwide Screening for Bee Viruses and Parasites in Belgian Honey Bees. Viruses.

[38] Lage V.M.G.B., Santana C.D., Patrocínio E., Noronha R.P., de Melo R.L., de Jesus Barbosa C., da Cunha Lima S.T. (2022). Prevalence of *Nosema Ceranae* in Apiculture Regions of Bahia State, Brazil. Ciência Rural.

[39] Bordin F., Zulian L., Granato A., Caldon M., Colamonico R., Toson M., Trevisan L., Biasion L., Mutinelli F. (2022). Presence of Known and Emerging Honey Bee Pathogens in Apiaries of Veneto Region (Northeast of Italy) during Spring 2020 and 2021. Appl. Sci..

[40] Naudi S., Šteiselis J., Jürison M., Raimets R., Tummeleht L., Praakle K., Raie A., Karise R. (2021). Variation in the Distribution of *Nosema* Species in Honeybees (*Apis Mellifera* Linnaeus) between the Neighboring Countries Estonia and Latvia. Vet. Sci..

[41] Imani Baran A., Kalami H., Mazaheri J., Hamidian G. (2022). *Vairimorpha Ceranae* Was the Only Detected Microsporidian Species from Iranian Honey Bee Colonies: A Molecular and Phylogenetic Study. Parasitol. Res..

[42] Martín-Hernández R., Meana A., Prieto L., Salvador A.M., Garrido-Bailón E., Higes M. (2007). Outcome of Colonization of *Apis Mellifera* by *Nosema Ceranae*. Appl. Environ. Microbiol..

[43] Klee J., Besana A.M., Genersch E., Gisder S., Nanetti A., Tam D.Q., Chinh T.X., Puerta F., Ruz J.M., Kryger P. (2007). Widespread Dispersal of the Microsporidian *Nosema Ceranae*, an Emergent Pathogen of the Western Honey Bee, *Apis Mellifera*. J. Invertebr. Pathol..

[44] Mederle N., Lobo M.L., Morariu S., Morariu F., Darabus G., Mederle O., Matos O. (2018). Microscopic and Molecular Detection of *Nosema Ceranae* in Honeybee *Apis Mellifera* L. from Romania. Rev. Chim..

[45] Wang Q., Dai P., Guzman-Novoa E., Wu Y., Hou C., Diao Q. (2019). *Nosema Ceranae*, the Most Common Microsporidium Infecting *Apis Mellifera* in the Main Beekeeping Regions of China since at Least 2005. J. Apic. Res..

[46] Traver B.E., Fell R.D. (2011). Prevalence and Infection Intensity of *Nosema* in Honey Bee (*Apis Mellifera* L.) Colonies in Virginia. J. Invertebr. Pathol..

[47] Gisder S., Schüler V., Horchler L.L., Groth D., Genersch E. (2017). Long-Term Temporal Trends of *Nosema* Spp. Infection Prevalence in Northeast Germany: Continuous Spread of *Nosema Ceranae*, an Emerging Pathogen of Honey Bees (*Apis Mellifera*), but No General Replacement of *Nosema Apis*. Front. Cell. Infect. Microbiol..

[48] Hall R.J., Pragert H., Phiri B.J., Fan Q.H., Li X., Parnell A., Stanislawek W.L., McDonald C.M., Ha H.J., McDonald W. (2021). Apicultural Practice and Disease Prevalence in *Apis Mellifera*, New Zealand: A Longitudinal Study. J. Apic. Res..

[49] Antúnez K., Anido M., Branchiccela B., Harriet J., Campa J., Invernizzi C., Santos E., Higes M., Martín-Hernández R., Zunino P. (2015). Seasonal Variation of Honeybee Pathogens and Its Association with Pollen Diversity in Uruguay. Microb. Ecol..

[50] Traver B.E., Williams M.R., Fell R.D. (2012). Comparison of within Hive Sampling and Seasonal Activity of *Nosema Ceranae* in Honey Bee Colonies. J. Invertebr. Pathol..

[51] Tentcheva D., Gauthier L., Zappulla N., Dainat B., Cousserans F., Colin M.E., Bergoin M. (2004). Prevalence and seasonal variations of six bee viruses in *Apis mellifera* L. and *Varroa destructor* mite populations in France. Appl. Environ. Microbiol..

[52] Gauthier L., Tentcheva D., Tournaire M., Dainat B., Cousserans F., Colin M.E., Bergoin M. (2007). Viral load estimation in asymptomatic honey bee colonies using the quantitative RT-PCR technique. Apidologie.

[53] Direção Regional da Agricultura (DRA) (2022). Programa Sanitário Apícola—Região Autónoma dos Açores.

[54] Direção Regional da Agricultura (DRA) (2021). Anexo I—PSA 2021—Situação Epidemiológica (2016–2021).

[55] Lopes A.R., Martín-Hernández R., Higes M., Segura S.K., Henriques D., Pinto M.A. (2022). Colonisation Patterns of *Nosema Ceranae* in the Azores Archipelago. Vet. Sci..

[56] Henriques D., Lopes A.R., Chejanovsky N., Dalmon A., Higes M., Jabal-Uriel C., Le Conte Y., Reyes-Carreño M., Soroker V., Martín-Hernández R. (2022). Mitochondrial and Nuclear Diversity of Colonies of Varying Origins: Contrasting Patterns Inferred from the Intergenic TRNAleu-Cox2 Region and Immune SNPs. J. Apic. Res..

[57] Urbieta-Magro A., Higes M., Meana A., Gómez-Moracho T., Rodríguez-García C., Barrios L., Martín-Hernández R. (2019). The Levels of Natural *Nosema* Spp. Infection in *Apis Mellifera Iberiensis* Brood Stages. Int. J. Parasitol..

[58] Delaplane K.S., Van Der Steen J., Guzman-Novoa E. (2013). Standard Methods for Estimating Strength Parameters of *Apis Mellifera* Colonies. J. Apic. Res..

[59] Dietemann V., Nazzi F., Martin S.J., Anderson D.L., Locke B., Delaplane K.S., Wauquiez Q., Tannahill C., Frey E., Ziegelmann B. (2013). Standard Methods for *Varroa* Research. J. Apic. Res..

[60] Ozgor E., Guzerin E., Keskin N. (2015). Determination and Comparison of Nosema Apis and Nosema Ceranae in Terms of Geographic and Climatic Factors. Hacet. J. Biol. Chem..

[61] Vejnovic B., Stevanovic J., Schwarz R.S., Aleksic N., Mirilovic M., Jovanovic N.M., Stanimirovic Z. (2017). Quantitative PCR Assessment of *Lotmaria Passim* in *Apis Mellifera* Colonies Co-Infected Naturally with *Nosema Ceranae*. J. Invertebr. Pathol..

[62] Botías C., Martín-Hernández R., Barrios L., Meana A., Higes M. (2013). *Nosema* Spp. Infection and Its Negative Effects on Honey Bees (*Apis Mellifera Iberiensis*) at the Colony Level. Vet. Res..

[63] Goblirsch M., Huang Z.Y., Spivak M. (2013). Physiological and Behavioral Changes in Honey Bees (*Apis Mellifera*) Induced by *Nosema Ceranae* Infection. PLoS ONE.

[64] Muñoz I., Cepero A., Pinto M.A., Martín-Hernández R., Higes M., De la Rúa P. (2014). Presence of *Nosema Ceranae* Associated with Honeybee Queen Introductions. Infect. Genet. Evol..

[65] Botías C., Martín-Hernández R., Días J., García-Palencia P., Matabuena M., Juarranz Á., Barrios L., Meana A., Nanetti A., Higes M. (2012). The Effect of Induced Queen Replacement on *Nosema* Spp. Infection in Honey Bee (*Apis Mellifera Iberiensis*) Colonies. Environ. Microbiol..

[66] Simeunovic P., Stevanovic J., Cirkovic D., Radojicic S., Lakic N., Stanisic L., Stanimirovic Z. (2014). *Nosema Ceranae* and Queen Age Influence the Reproduction and Productivity of the Honey Bee Colony. J. Apic. Res..

[67] Botías C., Martín-Hernández R., Meana A., Higes M. (2013). Screening Alternative Therapies to Control Nosemosis Type C in Honey Bee (*Apis Mellifera Iberiensis*) Colonies. Res. Vet. Sci..

[68] Mariani F., Maggi M., Porrini M., Fuselli S., Caraballo G., Brasesco C., Barrios C., Principal J., Martin E. (2012). Parasitic Interactions between *Nosema* Spp. and *Varroa Destructor* in *Apis Mellifera* Colonies. Zootec. Trop..

[69] Flores J.M., Gámiz V., Jiménez-Marín Á., Flores-Cortés A., Gil-Lebrero S., Garrido J.J., Hernando M.D. (2021). Impact of *Varroa Destructor* and Associated Pathologies on the Colony Collapse Disorder Affecting Honey Bees. Res. Vet. Sci..

[70] Nanetti A., Rodriguez-García C., Meana A., Martín-Hernández R., Higes M. (2015). Effect of Oxalic Acid on *Nosema Ceranae* Infection. Res. Vet. Sci..

[71] Botías C., Martín-Hernández R., Barrios L., Garrido-Bailón E., Nanetti A., Meana A., Higes M. (2012). *Nosema* Spp. Parasitization Decreases the Effectiveness of Acaricide Strips (Apivar ^®^) in Treating Varroosis of Honey Bee (*Apis Mellifera Iberiensis*) Colonies. Environ. Microbiol. Rep..

[72] Doublet V., Natsopoulou M.E., Zschiesche L., Paxton R.J. (2015). Within-Host Competition among the Honey Bees Pathogens *Nosema Ceranae* and Deformed Wing Virus Is Asymmetric and to the Disadvantage of the Virus. J. Invertebr. Pathol..

[73] Little C.M., Shutler D., Williams G.R. (2016). Associations among *Nosema* Spp. Fungi, *Varroa Destructor* Mites and Chemical Treatments in Honey Bees, *Apis Mellifera*. J. Apic. Res..

[74] Gray A., Adjlane N., Arab A., Ballis A., Brusbardis V., Charrière J.D., Chlebo R., Coffey M.F., Cornelissen B., Amaro da Costa C. (2020). Honey Bee Colony Winter Loss Rates for 35 Countries Participating in the COLOSS Survey for Winter 2018–2019, and the Effects of a New Queen on the Risk of Colony Winter Loss. J. Apic. Res..

[75] Villa J.D., Bourgeois A.L., Danka R.G. (2013). Negative Evidence for Effects of Genetic Origin of Bees on *Nosema Ceranae*, Positive Evidence for Effects of *Nosema Ceranae* on Bees. Apidologie.

[76] Bekele A.Z., Mor S.K., Phelps N.B.D., Goyal S.M., Armién A.G. (2015). A Case Report of *Nosema Ceranae* Infection in Honey Bees in Minnesota, USA. Vet. Q..

[77] Hatjina F., Tsoktouridis G., Bouga M., Charistos L., Evangelou V., Avtzis D., Meeus I., Brunain M., Smagghe G., De Graaf D.C. (2011). Polar Tube Protein Gene Diversity among *Nosema Ceranae* Strains Derived from a Greek Honey Bee Health Study. J. Invertebr. Pathol..

[78] Betti M.I., Wahl L.M., Zamir M. (2014). Effects of Infection on Honey Bee Population Dynamics: A Model. PLoS ONE.

[79] Naug D. (2014). Infected Honeybee Foragers Incur a Higher Loss in Efficiency than in the Rate of Energetic Gain. Biol. Lett..

[80] Khoury D.S., Barron A.B., Myerscough M.R. (2013). Modelling Food and Population Dynamics in Honey Bee Colonies. PLoS ONE.

[81] Dainat B., Evans J.D., Chen Y.P., Gauthier L., Neumann P. (2012). Predictive Markers of Honey Bee Colony Collapse. PLoS ONE.

[82] Alonso-Prados E., González-Porto A.V., Bernal J.L., Bernal J., Martín-Hernández R., Higes M. (2021). A Case Report of Chronic Stress in Honey Bee Colonies Induced by Pathogens and Acaricide Residues. Pathogens.

[83] Aufauvre J., Biron D.G., Vidau C., Fontbonne R., Roudel M., Diogon M., Viguès B., Belzunces L.P., Delbac F., Blot N. (2012). Parasite-Insecticide Interactions: A Case Study of *Nosema Ceranae* and Fipronil Synergy on Honeybee. Sci. Rep..

[84] Dussaubat C., Maisonnasse A., Crauser D., Tchamitchian S., Bonnet M., Cousin M., Kretzschmar A., Brunet J.L., Le Conte Y. (2016). Combined Neonicotinoid Pesticide and Parasite Stress Alter Honeybee Queens’ Physiology and Survival. Sci. Rep..

[85] Beaurepaire A., Piot N., Doublet V., Antunez K., Campbell E., Chantawannakul P., Chejanovsky N., Gajda A., Heerman M., Panziera D. (2020). Diversity and Global Distribution of Viruses of the Western Honey Bee, Apis Mellifera. Insects.

[86] Coulon M., Dalmon A., Di Prisco G., Prado A., Arban F., Dubois E., Ribière-Chabert M., Alaux C., Thiéry R., Le Conte Y. (2020). Interactions Between Thiamethoxam and Deformed Wing Virus Can Drastically Impair Flight Behavior of Honey Bees. Front. Microbiol..

[87] Di Prisco G., Cavaliere V., Annoscia D., Varricchio P., Caprio E., Nazzi F., Gargiulo G., Pennacchio F. (2013). Neonicotinoid Clothianidin Adversely Affects Insect Immunity and Promotes Replication of a Viral Pathogen in Honey Bees. Proc. Natl. Acad. Sci. USA.

[88] Nazzi F., Brown S.P., Annoscia D., Del Piccolo F., Di Prisco G., Varricchio P., Vedova G.D., Cattonaro F., Caprio E., Pennacchio F. (2012). Synergistic Parasite-Pathogen Interactions Mediated by Host Immunity Can Drive the Collapse of Honeybee Colonies. PLoS Pathog..

[89] Martin S.J., Hardy J., Villalobos E., Martín-Hernández R., Nikaido S., Higes M. (2013). Do the Honeybee Pathogens *Nosema Ceranae* and Deformed Wing Virus Act Synergistically?. Environ. Microbiol. Rep..

[90] Ryabov E.V., Wood G.R., Fannon J.M., Moore J.D., Bull J.C., Chandler D., Mead A., Burroughs N., Evans D.J. (2014). A Virulent Strain of Deformed Wing Virus (DWV) of Honeybees (*Apis Mellifera*) Prevails after *Varroa Destructor*-Mediated, or In Vitro, Transmission. PLoS Pathog..

[91] Moore J., Jironkin A., Chandler D., Burroughs N., Evans D.J., Ryabov E.V. (2011). Recombinants between Deformed Wing Virus and *Varroa Destructor* Virus-1 May Prevail in *Varroa Destructor*-Infested Honeybee Colonies. J. Gen. Virol..

[92] Bourgeois A.L., Rinderer T.E., Sylvester H.A., Holloway B., Oldroyd B.P. (2012). Patterns of *Apis Mellifera* Infestation by *Nosema Ceranae* Support the Parasite Hypothesis for the Evolution of Extreme Polyandry in Eusocial Insects. Apidologie.

[93] Lodesani M., Costa C., Besana A., Dall’Olio R., Franceschetti S., Tesoriero D., Vaccari G. (2014). Impact of Control Strategies for *Varroa Destructor* on Colony Survival and Health in Northern and Central Regions of Italy. J. Apic. Res..

