# Supplementary material for: Epidemiology of the Microsporidium Nosema ceranae in Four Mediterranean Countries
Source: Insects. 2022 Sep 16;13(9):844. doi: 10.3390/insects13090844 (PMC9505483; doi:10.3390/insects13090844)
Supplement: Supplementary file 1 [file insects-13-00844-s001.zip › Supplementary Table S1.pdf]

Supplementary Table S1. Mean levels of infection per month during the study period in each apiary. SMI apiary is not included due to its absence of *N. ceranae* detection.

CIAPA	2018					2019				
	Feb	Apr	Jun	Aug	Oct	Feb	Apr	Jun	Aug	Oct
Mean	16.00	27.64	5.52	10.59	7.75	7.50	14.00	15.14	10.77	15.69
Median	12.00	22.00	4.00	8.00	4.00	6.00	12.00	16.00	8.00	12.00
Std. Dev.	17.37	23.74	8.05	7.48	7.93	6.67	13.39	6.87	7.37	10.13
Min	0.00	4.00	0.00	0.00	0.00	0.00	0.00	4.00	0.00	4.00
Max	60.00	96.00	36.00	28.00	24.00	24.00	52.00	28.00	24.00	32.00
Total	15	22	21	17	16	16	16	14	13	13

INRA	2018					2019				
	Feb	Apr	Jun	Aug	Oct	Feb	Apr	Jun	Aug	Oct
Mean	0.96	0.70	0.57	1.70	1.33	0.40	0.00	4.00	3.60	0.00
Median	0.00	0.00	0.00	0.00	0.00	0.00	0.00	0.00	2.00	0.00
Std. Dev.	3.47	1.71	1.51	4.16	2.83	1.26	0.00	7.06	4.40	0.00
Min	0.00	0.00	0.00	0.00	0.00	0.00	0.00	0.00	0.00	0.00
Max	12.50	4.20	4.00	13.00	8.00	4.00	0.00	20.00	12.00	0.00
Total	13	6	7	10	9	10	10	10	10	10

OUE	2018				2019			
	Apr	Jun	Aug	Oct	Apr	Jun	Aug	Oct
Mean	28.42	27.67	14.40	1.20	0.80	8.80	2.00	5.60
Median	20.33	32.00	16.00	0.00	0.00	6.00	0.00	0.00
Std. Dev.	22.34	12.78	11.50	1.93	1.69	7.50	3.89	14.99
Min	8.00	4.00	0.00	0.00	0.00	0.00	0.00	0.00
Max	70.83	41.67	32.00	4.00	4.00	24.00	12.00	48.00
Total	10	10	10	10	10	10	10	10

ARO	2018				2019			
	Feb	Apr	Jun	Oct	Feb	Apr	Jun	Oct
Mean	47.00	29.60	48.50	16.11	10.00	13.18	49.55	56.00
Median	51.00	30.00	47.50	5.00	10.00	5.00	55.00	52.50
Std. Dev.	24.71	10.19	25.17	18.33	8.82	17.50	14.91	15.95
Min	0.00	15.00	0.00	0.00	0.00	0.00	25.00	35.00
Max	80.00	50.00	85.00	45.00	25.00	50.00	75.00	90.00
Total	12	10	10	9	10	11	11	10

CIMO	2018					2019				
	Feb	Apr	Jun	Aug	Oct	Feb	Apr	Jun	Aug	Oct
Mean	16.56	51.05	10.64	10.75	14.04	24.35	19.75	7.64	11.13	4.85
Median	16.00	48.00	8.70	4.17	8.70	26.00	16.25	0.00	8.00	0.00
Std. Dev.	11.48	15.82	8.04	11.95	13.86	13.35	20.54	14.25	11.03	10.41
Min	0.00	24.00	0.00	0.00	0.00	4.17	0.00	0.00	0.00	0.00
Max	44.00	79.17	36.00	40.00	52.00	48.00	76.00	48.00	33.33	32.00
Total	15	13	15	15	15	12	12	11	11	11
